# Comprehensive pan-cancer analysis and experimental validation reveal FCHSD1 as a potential biomarker for diagnosis, immune infiltration, and prognosis

**DOI:** 10.3389/fonc.2025.1547067

**Published:** 2025-06-04

**Authors:** Ming Liu, Guixin Ding, Gonglin Tang, Shangjing Liu, Qiancheng Mao, Xidong Wang, Qingsong Zou, Jitao Wu

**Affiliations:** ^1^ Second Clinical Medical College, Binzhou Medical University, Yantai, Shandong, China; ^2^ Department of Urology, Yantai Yuhuangding Hospital, Qingdao University, Yantai, Shandong, China

**Keywords:** FCHSD1, expression profile, prognosis, immune infiltration, pan-cancer

## Abstract

**Background:**

FCHSD1 is a member of the F-BAR family containing one amino terminal F-BAR domain and two SH3 domains. At present, there are no relevant pan-cancer comprehensive studies on the predictive potential and immune infiltration of FCHSD1 for cancer.

**Methods:**

FCHSD1 expression profiles were analyzed through the use of various tools, including TIMER, GEPIA, R packages, and the UALCAN database. The genetic alteration status of FCHSD1 in human pan-cancer was studied using the cBioPortal website. The effect of FCHSD1 on immune infiltration was examined using the TIMER and TISIDB databases. We confirmed the association between FCHSD1 expression and patient prognosis using survival analysis from GEPIA and R packages. The drug database was utilized to analyze the sensitivity of FCHSD1 to drugs. The FCHSD1 interactive genes were obtained through the STRING and GeneMANIA platforms, respectively, and analyzed by GO and KEGG. The expression and function of FCHSD1 in renal cancer cells and tissues have also been biologically validated *in vitro*.

**Results:**

FCHSD1 expression was found to be elevated in tumor tissues compared to adjacent tissues. The expression of FCHSD1 varied across different clinical stages, pathological stages, immune types, and molecular subtypes. Higher expression of FCHSD1 predicts worse outcomes for several cancer types, such as CHOL and KIRC. High FCHSD1 expression was positively correlated with immune cell infiltration in different cancer types. Additionally, the FCHSD1 co-expression gene network may be involved in endocytosis. *In vitro* experiments revealed that the expression of FCHSD1 in renal cancer cells and tissues was higher than that in normal cells and adjacent non-cancerous tissues. Functional assays revealed that FCHSD1 knockdown significantly suppressed proliferation and migration in ACHN and 769P cells.

**Conclusion:**

FCHSD1 has the potential to serve as a prognostic and immunological marker for pan-cancer, and may also be a crucial target for future immunotherapy.

## Introduction

Cancer represents one of the most formidable global public health challenges, posing a significant threat to human health and survival (1). This disease imposes a significant global health and economic burden. Current therapeutic strategies for cancer primarily include surgery, chemotherapy, radiotherapy, targeted therapy, and immunotherapy (2). Although these therapies have shown efficacy in cancer treatment, patient prognosis remains suboptimal due to drug resistance and side effects. In recent years, targeted therapies have demonstrated remarkable progress in improving treatment response rates, prolonging progression-free survival, reducing treatment-related toxicity, and decreasing cancer-specific mortality. However, due to tumor heterogeneity and the development of multiple drug resistance mechanisms, the clinical benefits of targeted therapies remain limited, with suboptimal improvements in long-term patient outcomes. In this context, the identification of clinically relevant biomarkers is of paramount importance for facilitating early diagnosis, accurate prognostic assessment, and optimization of treatment strategies.

In 2004, Masuko Katoh et al. identified and characterized the FCHSD1 and FCHSD2 genes. Previous studies have identified FCHSD1 as a member of the F-BAR family with two SH3 domains (3). F-BAR protein, also known as Pombe Cdc15 homology (PCH) protein family, generally has a Fes/CIP4 homology (FCH) domain at its N-terminus, or the F-BAR domain, and one or more SH3 domains at its C-terminus (4). F-BAR proteins play a bridging role between cytoskeleton-associated proteins and plasma membranes in mammalian cells (5). The F-BAR protein typically contains the SH3 domain, which mediates multiple interactions with endocytosis, cytoskeleton, and signaling proteins (6–8). Many studies have shown that some members of the F-BAR protein family are associated with neurological diseases, chronic obstructive pulmonary disease (COPD) and diseases such as tumors (9–12). To sum up, proteins containing F-BAR/SH3 may be associated with the development and development of cancer by regulating molecular trafficking.

The tumor microenvironment (TME) is primarily composed of blood vessels cells, immune cells, fibroblasts, stromal cells, and extracellular matrix (13). Numerous studies have revealed the crucial role of the TME in tumor initiation and progression (14). The immune infiltration plays a paramount role in shaping the immune microenvironment (15). The immune system exerts surveillance and inhibitory functions against tumor development, yet tumors can evade immune responses by suppressing the microenvironment. Immunotherapy, as a novel approach to cancer treatment, can remodel the immune microenvironment to sustain tumor surveillance, cytotoxicity, and inhibition of tumor growth (16). Therefore, the quest for reliable tumor immune-related biomarkers as targets for cancer immunotherapy is of utmost importance.

However, there is no systematic analysis of FCHSD1 in pan-carcinoma. In this study, we systematically analyzed the difference in FCHSD1 expression in pan-carcinoma, and the association of FCHSD1 expression for cancer clinical stage, molecular typing, immune cell infiltration, molecular function, prognosis, drug sensitivity, etc. Finally, we also explore the role of FCHSD1 in genitourinary cancer, and verify its role in human pan-carcinoma through vitro experiments. These findings showed that FCHSD1 may be a potential novel biomarker. This study can provide a new and unique perspective for the diagnosis, clinical treatment and prognosis analysis of cancer in the future.

## Methods

### The Cancer Genome Atlas database

The TCGA database (https://tcga-data.nci.nih.gov/tcga/), a freely accessible online repository of cancer genomes, encompasses a vast array of clinical and pathological data pertaining to over 30 distinct cancer types. Using this extensive dataset, we analyzed RNA-seq expression, prognosis, and clinicopathological information in pan-cancer patients.

### Tumor Immune Estimation Resource database

Tumor Immune Estimation Resource (TIMER, https://cistrome.shinyapps.io/timer/) is a comprehensive resource site for systematic analysis of immune infiltration in different cancer types (17). We used TIMER’s Diff Exp module to study the differential expression of FCHSD1 in TCGA tumor tissues and adjacent normal tissues. The statistical significance of this differential expression was assessed using Wilcoxon’s test. We then used the Gene module to visualize the correlation between FCHSD1 expression and the level of immune invasion in different cancer types, and showed the rho value of the purity-corrected partial Spearman and its statistical significance.

### UALCAN, and clinical proteomic tumor analysis consortium

UALCAN (http://ualcan.path.uab.edu) is an interactive portal for in-depth analysis using TCGA RNA-seq and clinical data from 31 cancer types (18). Based on data from the Clinical Proteomics Consortium for Tumor Analysis (CPTAC) dataset, we explored the protein expression levels of FCHSD1 between tumors and normal tissues. The significance of differences was assessed using Student’s *t*-test, and *p* < 0.05 was considered statistically significant. Multiple testing correction was not applied.

### The Gene Expression Profiling Interactive Analysis analysis

GEPIA (http://gepia.cancer-pku.cn/index.html) is an online platform containing RNA sequencing expression data from tumor and normal samples from the TCGA and GTEx projects (19). FCHSD1 expression at different tumor stages in pan-carcinoma was compared using this platform. The effect of FCHSD1 expression on prognosis (overall survival and disease-free survival) was also studied. In addition, we used GEPIA to search for the first 100 FCHSD1-related genes derived from all TCGA tumor tissues and corresponding normal tissues.

### cBioPortal

The cBioPortal database is an open-access web-based resource that includes molecular profiles and clinical properties from the Cancer Genome Atlas (20, 21). We used it to explore and analyze the genetic alteration of FCHSD1 in pan-carcinoma.

### Protein-Protein Interaction network and functional enrichment analysis

The STRING database is a website that predicts physical and functional associations between proteins (22, 23). 50 interaction proteins of FCHSD1 were predicted using STRING. GeneMANIA (http://www.genemania.org) is a website that establishes input gene protein-protein interaction (PPI) networks from large amounts of functional association data, which can provide gene function prediction hypotheses and identify genes with comparable effects (24). We used GeneMANIA to build and analyze FCHSD1’s PPI network. We utilized the ClusterProfiler package to conduct Gene Ontology (GO) enrichment and Kyoto Encyclopedia of Genes and Genomes (KEGG) pathway analysis of coexpression genes. The results were visualized using “ggplot2”.

### Tumor-Immune System Interaction Database

TISIDB (http://cis.hku.hk/TISIDB/index.php) integrates genomics, transcriptomics and clinical data of 30 cancer types from The Cancer Genome Atlas (TCGA) as well as other data for tumor and immune system interactions (25). TISIB was used to evaluate FCHSD1 expression in different immune subtypes and molecular subtypes of tumors. We also used TISIB to explore the relationship between FCHSD1 and immune infiltration, MHC expression, immune inhibitors, immunostimulators, chemokines and chemokine receptors.

### Sample collection

Twenty eligible ccRCC patients were enrolled in this study after receiving approval from the ethics committee of the affiliated Yantai Yuhuangding Hospital of Qingdao University. Pathological specimens were obtained from patients who underwent radical or partial nephrectomy, and were confirmed by two independent pathologists.

### *In vitro* cell culture and siRNA transfection

The normal human renal epithelial cell line (HK-2) and KIRC cell lines (ACHN, A498, 786O, 769P, and Caki) used in the *in vitro* experiments were purchased from the Cell Bank of the Chinese Academy of Sciences. The cell lines were cultivated according to their instructions. All siRNAs were purchased from Sangon Biotech (Shanghai) and transfected into the selected cell lines following the manufacturer’s instructions. The negative control used was the company’s universal negative control. The sequences of the three siRNA-FCHSD1 are as follows: FCHSD1-1-sense: 5’-GCAAUGAGUACCUGCUUAAdTdT-3’; FCHSD1-1-antisense: 5’-UUAAGCAGGUACUCAUUGCdTdT-3’; FCHSD1-2-sense: 5’-GCUGGGAGCAAGACCUGAAdTdT-3’; FCHSD1-2-antisense: 5’-UUCAGGUCUUGCUCCCAGCdTdT-3’; FCHSD1-3-sense: 5’-GUGACUACAAGAUCCAGAAdTdT-3’; FCHSD1-3-antisense: 5’-UUCUGGAUCUUGUAGUCACdTdT-3’.

### Westen blot

Total proteins from the desired cell lines and tissues were extracted using Radio ImmunoPrecipitation Assay (RIPA) lysis buffer. Electrophoresis was performed on 10% SDS-PAGE gels, followed by transfer to polyvinylidene fluoride (PVDF) membranes. The PVDF membranes were blocked using a rapid blocking solution and then incubated overnight at 4°C with rabbit polyclonal primary antibodies against FCHSD1 (Proteintech) diluted at a ratio of 1:1000. The next day, the membranes were washed five times with Tris-buffered saline containing Tween-20 (TBST). After removing the primary antibodies, the membranes were incubated with secondary antibodies for 1 hour at room temperature. Subsequently, the membranes were washed five more times with TBST. Finally, the membranes were developed using a luminescent solution.

### RNA extraction and quantitative real-time PCR

RNA was extracted using Trizol reagent (Pufei, Shanghai) as per the manufacturer’s guidelines, and converted to cDNA using the Promega M-MLV kit. The qPCR primer sequence for FCHSD1 was as follows: forward 5′- GCCTGGAGAAAGAGGTTCAGCG-3′ and reverse 5′- CCTCTGTTCTATGCTTGGAGC-3′.

### Cell counting kit-8 assay

Cell proliferation was assessed using the CCK-8 assay. Cells were seeded into 96-well plates at a density of 5 × 10^3^ cells/well. Absorbance values at 450 nm were measured daily for 3 days according to the manufacturer’s instructions.

### Wound healing assay

Cells were seeded in 6-well plates and transfected with the aforementioned siRNAs when they reached 80-90% confluence. Forty-eight hours after transfection, a scratch was made across the center of each well using a 200 μL pipette tip. After washing with PBS, the medium was replaced with serum-free medium. Images of the wounds were captured at 0 h and 12 h post-scratching.

### Statistical analysis

Statistical analysis was performed using R (version 4.2.1) and the results were visualized using ggplot2 (version 3.3.3). To compare ccRCC tissues with normal surrounding tissues, we conducted the Mann-Whitney U test and paired t-test. GraphPad Prism 6 software was utilized for the statistical analysis. The statistical significance level was set at *p* ≤ 0.05 and denoted with * in the figure legends (*≤ 0.05; **< 0.01; ***< 0.001).

## Result

### Expression of FCHSD1 in multiple cancer tissues

**Figure 1:**
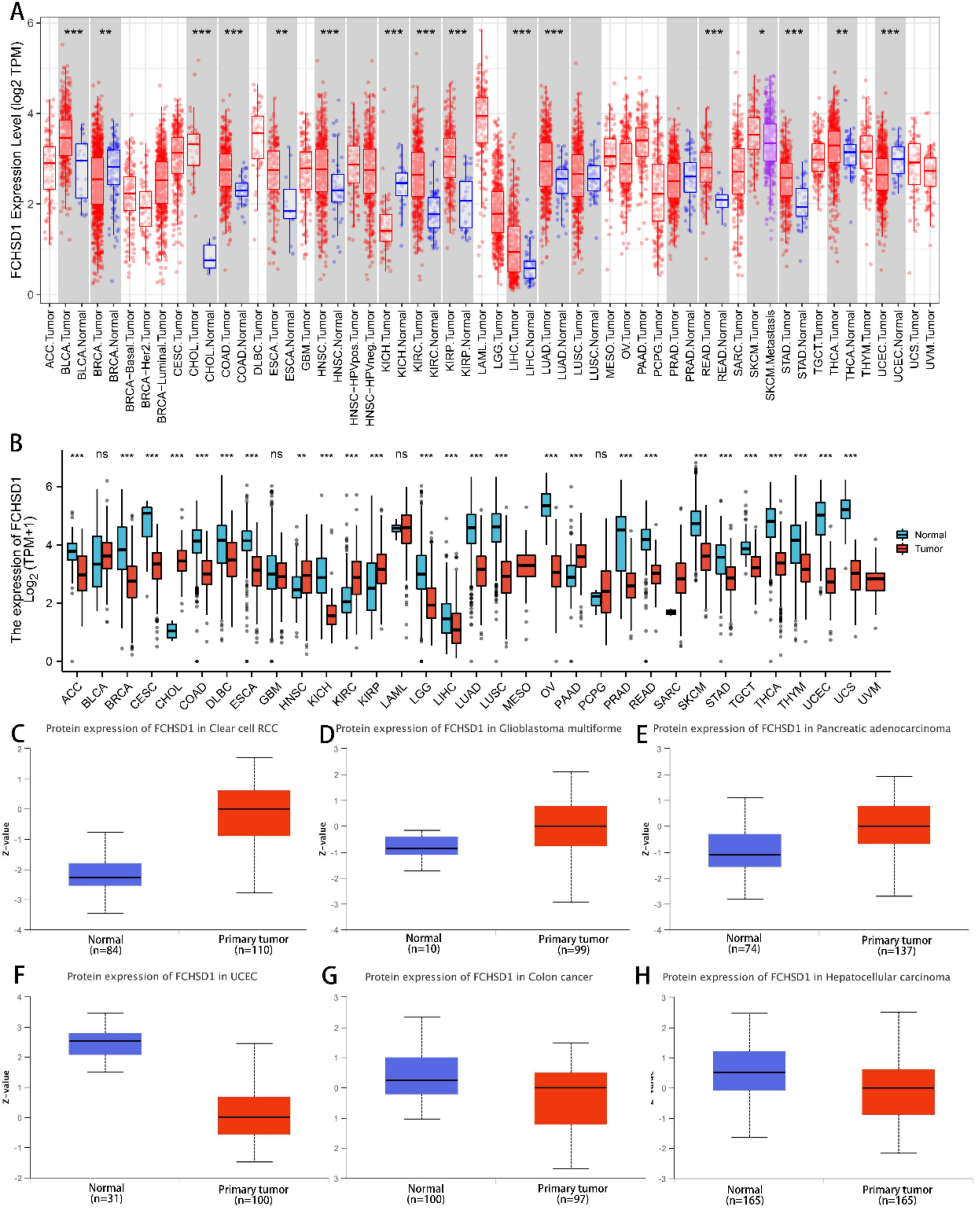
FCHSD1 expression is upregulated in most types of cancers compared with controls. **(A)** Exploration of FCHSD1 mRNA expression in human cancers and normal tissues using the TIMER database. **(B)** Comparison of FCHSD1 mRNA expression in human cancers using the R package. **(C-H)** FCHSD1 protein expression is changed in cancers examined using the UALCAN database. *p < 0.05, **p < 0.01, ***p < 0.001.

To determine differences in FCHSD1 expression across cancer types, several databases were used. Using data from the TCGA database, the expression of FCHSD1 in tumor tissue and normal controls was compared by TIMER. FCHSD1 has increased expression in 13 cancers, including bladder urothelial carcinoma (BLCA), cholangiocarcinoma (CHOL), colon adenocarcinoma (COAD), esophageal carcinoma (ESCA), head and neck squamous cell carcinoma (HNSC), renal clear cell carcinoma (KIRC), and renal papillary cell carcinoma (KIRP) (**Figure 1A**). Through further comparison of TCGA + GTEx database, it was found that FCHSD1 was significantly elevated in five tumor types: CHOL, HNSC, KIRC, KIRP, and pancreatic adenocarcinoma (PAAD) (**Figure 1B**). The CPTAC dataset of the UALCAN platform was used to further verify the expression of FCHSD1 protein in pan-carcinogenic tissues. FCHSD1 protein expression is reduced in uterine corpus endometrial carcinoma (UCEC), ovarian serous cystadenocarcinoma (OV) and liver hepatocellular carcinoma (LIHC) compared to normal tissue (**Figures 1C–E**). In contrast, FCHSD1 expression significant enhancement was found in KIRC, glioblastoma multiforme (GBM), PAAD (**Figures 1F–H**). These results suggest that FCHSD1 expression may play a key role in the progression of human tumors.

### FCHSD1 expression differentially correlates with tumor stages

**Figure 2:**
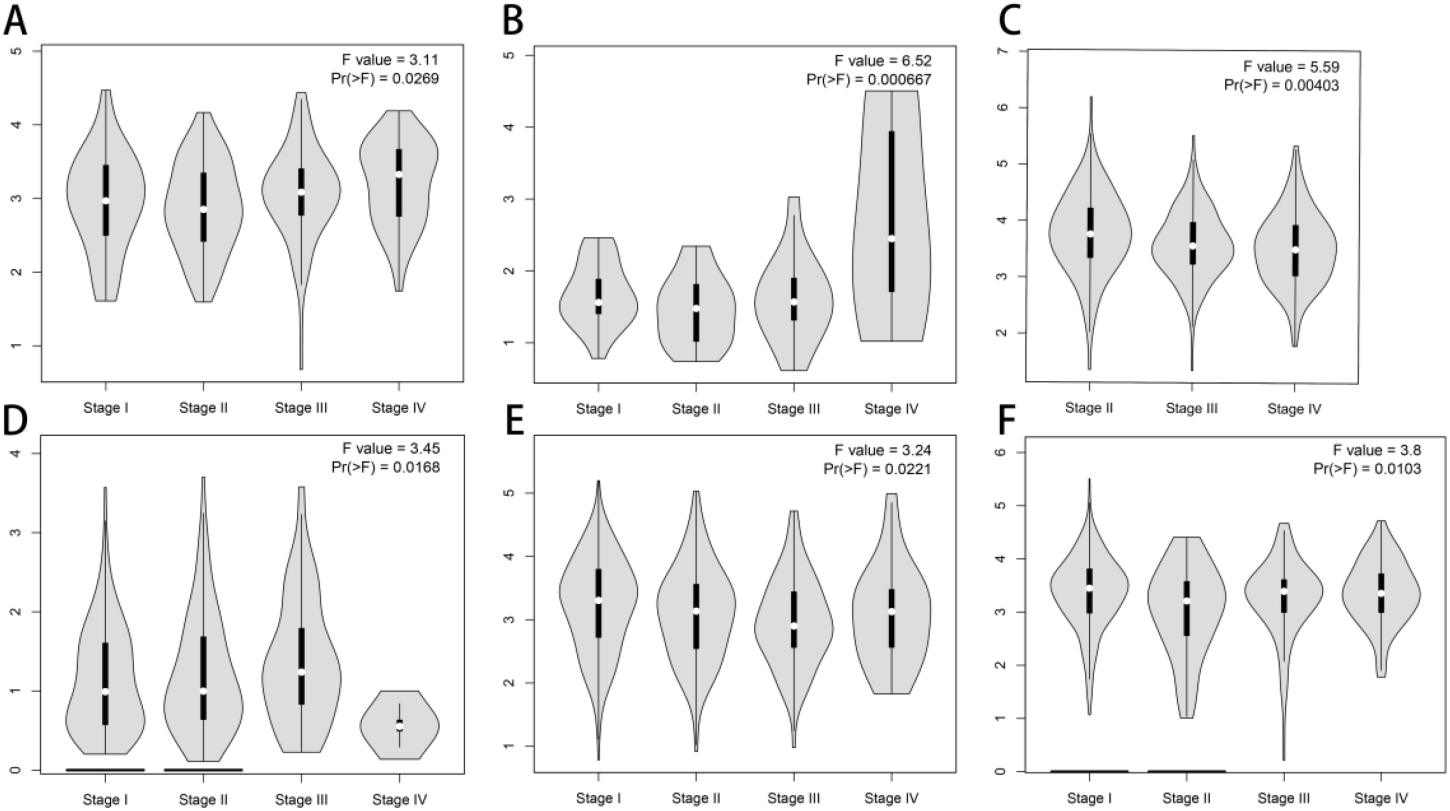
FCHSD1 expression is discriminately distributed in different stages and immune subtypes of cancers. FCHSD1 expression significantly varied across different tumor stages in COAD **(A)**, KICH **(B)**, BLCA **(C)**, LIHC **(D)**, LUAD **(E)** and THCA **(F)**.

To further explore FCHSD1 expression in human cancers obtained from different stages, we analyzed the relationship between FCHSD1 and tumor stages using GEPIA. In COAD, kidney chromophobe (KICH), stage IV tumor tissue was associated with higher expression of FCHSD1 compared to other stages (**Figures 2A, B**). However, FCHSD1 expression was decreased in stage IV of BLCA and LIHC tissues (**Figures 2C, D**). For lung adenocarcinoma (LUAD) and thyroid carcinoma (THCA), the correlation between the expression of FCHSD1 and staging was not obvious (**Figures 2E, F**).

### Genetic alteration analysis of FCHSD1

**Figure 3:**
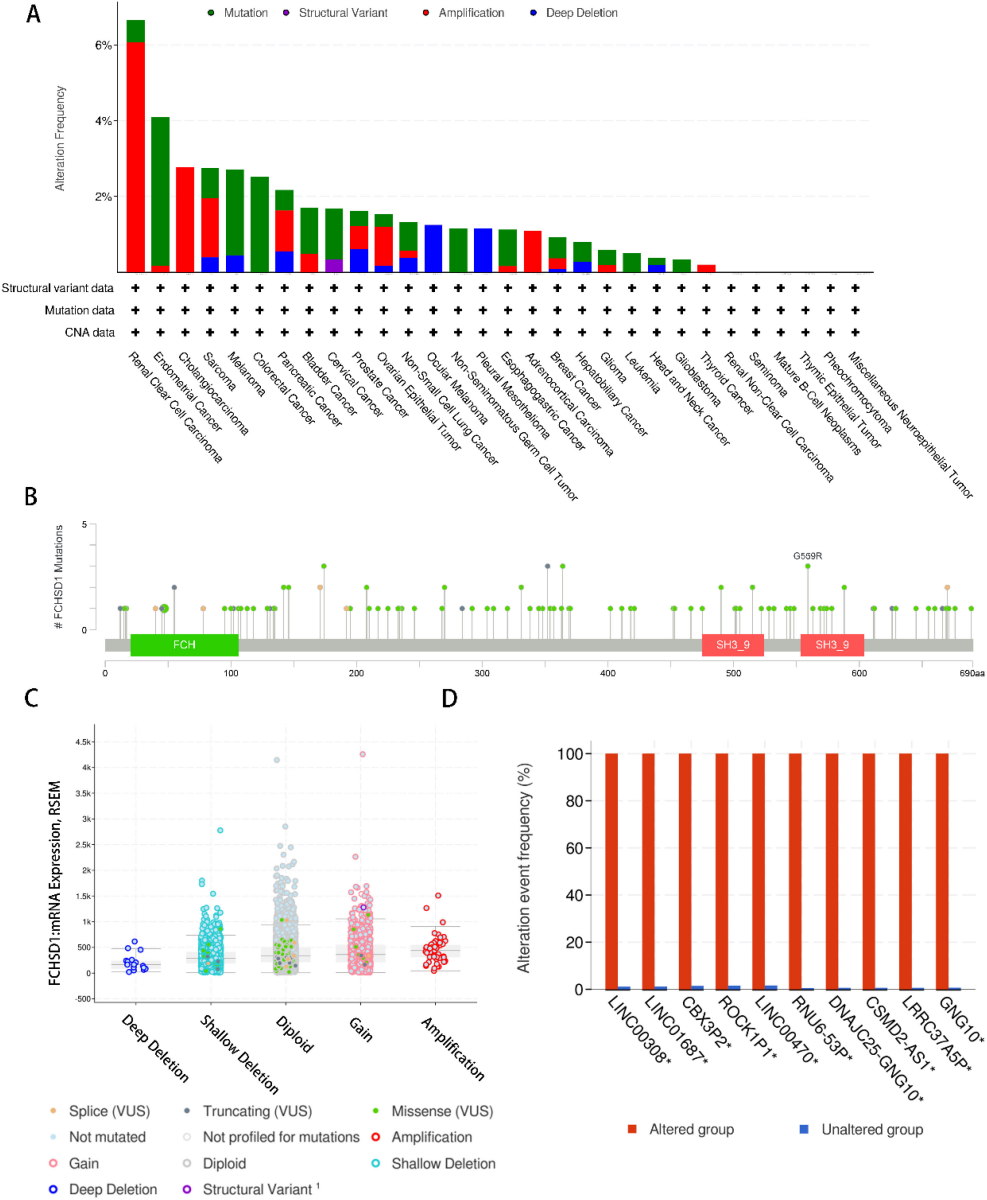
Genetic alteration analysis of FCHSD1 by cBioPortal. **(A)** Summary of the alteration frequency for mutation, structural variant, amplification, and deep deletion in various cancers. **(B)** Presentation of the types and sites of FCHSD1 genetic alteration. **(C)** The most common copy number change of FCHSD1 in cancers. **(D)** Genes with the highest frequency in any group.

To systematically elucidate the mutational characteristics and biological function of FCHSD1 in tumor progression, we used the cBioPortal website to study the genetic alteration status of FCHSD1 in human pan-carcinoma. Genetic alterations of FCHSD1 in tumors include mutations, structural variants, amplifications, and deep deletions. As shown, we observed that the highest frequency of FCHSD1 alterations was in patients with KIRC with “amplification” and “mutation” as the main alterations (6.07% + 0.59%). In cholangiocarcinoma, the genetic alterations of FCHSD1 are “amplification” (2.78%). In colorectal cancer, the genetic variation of FCHSD1 was “mutation” (4.35% and 2.53%) (**Figure 3A**). In addition, according to the cBioPortal database, missense mutations in FCHSD1 were found to be the main type of genetic alterations, and G559R alterations were detected in 2 cases of UCEC and 1 case of oligodendroglioma (**Figure 3B**). The most common copy number change of FCHSD1 is diploid, with shallow loss of proliferative function (**Figure 3C**). The frequency of genetically altered events in the altered group was higher in the altered group than in the unchanged group for LINC00308, LINC01687, CBX3P2, ROCK1P1, LINC00470, RNU6-53P, DNAJC25-GNG10, CSMD2-AS1, LRRC37A5P, and GNG10 (**Figure 3D**).

### FCHSD1 expression differentially correlates with immune subtypes and molecular subtypes

**Figure 4:**
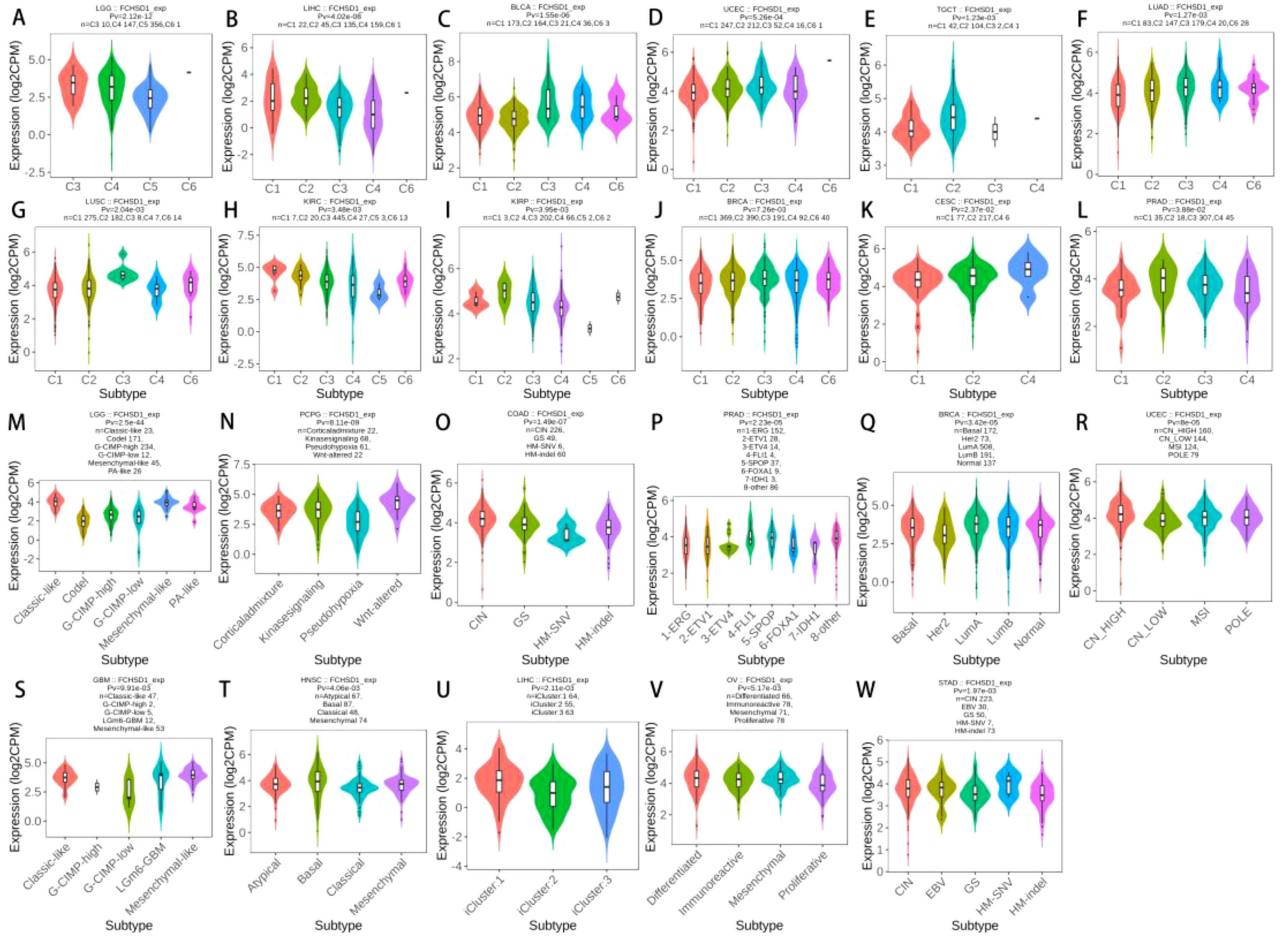
FCHSD1 expression is discriminately distributed in different immune subtypes and molecular subtypes of cancers. FCHSD1 expression in different immune subtypes of LGG **(A)**, LIHC **(B)**, BLCA **(C)**, UCEC **(D)**, TGCT **(E)**, LUAD **(F)**, LUSC **(G)**, KIRC **(H)**, KIRP **(I)**, BRCA **(J)**, CESC **(K)** and PRAD **(L)**. Variation of FCHSD1 expression was shown in LGG **(M)**, PCPG **(N)**, COAD **(O)**, PRAD **(P)**, BRCA **(Q)**, UCEC **(R)**, STAD **(S)**, LIHC **(T)**, HNSC **(U)**, OV **(V)** and GBM **(W)**.

The role of FCHSD1 expression in human cancer immunity and molecular subtypes is explored through the TISIDB website. Based on the transcriptomic characteristics of more than 10,000 patients in the Cancer Genome Atlas (TCGA) cancer type, the immunosubtypes of solid tumors are divided into six categories. The 6 types include C1 (wound healing), C2 (IFN-γ dominant), C3 (inflammation), C4 (lymphocyte depletion), C5 (immunocalm), C6 (TGF-β dominant). The results showed that the expression of FCHSD1 in LGG, LIHC, BLCA, UCEC, TGCT, LUAD, LUSC, KIRC, KIRP, BRCA, Cervical squamous cell carcinoma and endocervical adenocarcinoma (CESC), prostate adenocarcinoma (PRAD) was associated with different immune isotypes (**Figures 4A–L**). In addition, the expression of FCHSD1 also differs in different immune subtypes of one cancer type. We note that FCHSD1 is the lowest expressed in human cancers of C4 and C5 subtypes, and specifically in LGG, LIHC, LUSC, KIRC, KIRP, PRAD. The expression of FCHSD1 also differs in different molecular subtypes of tumors. FCHSD1 expression was significantly altered in 11 cancer types. For cancers of different molecular subtypes, significant associations in FCHSD1 expression were shown in 11 cancer types, including LGG, pheochromocytoma and paraganglioma (PCPG), COAD, PRAD, BRCA, UCEC, stomach adenocarcinoma (STAD), LIHC, HNSC, OV, and GBM (**Figures 4M–W**). Based on the above results, we conclude that FCHSD1 expression differs in pan-carcinoma of different immune isotypes and molecular subtypes.

### Correlation between immune infiltration and FCHSD1 expression

**Figure 5:**
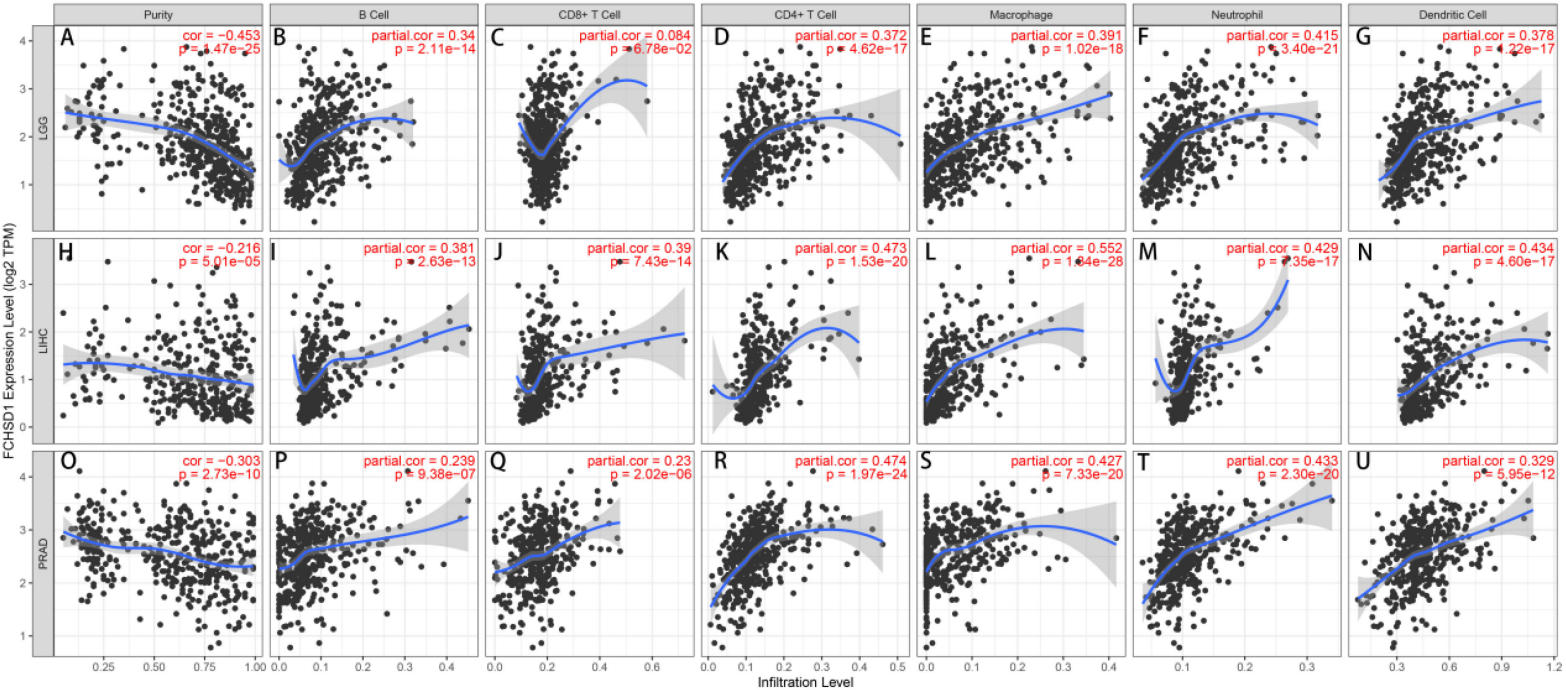
FCHSD1 expression is correlated with immune infiltration in cancers as analyzed using the TIMER database. FCHSD1 expression negatively correlated with tumor purity in LGG **(A)**, LIHC **(H)**, PRAD **(O)**. In LGG, FCHSD1 expression positively correlated with infiltration of B cell **(B)**, CD4 T cell **(D)**, macrophage **(E)**, neutrophil **(F)**, and dendritic cell **(G)**; However, FCHSD1 expression had no significant correlation with infiltration of CD8 T cell in LGG **(C)**; In LIHC, FCHSD1 expression positively correlated with infiltration of B cell **(I)**, CD8 T cell **(J)**, CD4 T cell **(K)**, macrophage **(L)**, neutrophil **(M)**, and dendritic cell **(N)**; In PRAD, FCHSD1 expression positively correlated with infiltration of B cell **(P)**, CD8 T cell **(Q)**, CD4 T cell **(R)**, macrophage **(S)**, neutrophil **(T)**, and dendritic cell **(U)**. The correlation coefficients with p-values are annotated in the figure.

Tumor-infiltrating immune cells are key players in tumor progression and can be studied to develop strategies for tumor immune response. The TIMER database was used to elucidate whether FCHSD1 affects immune infiltration in human cancer. The results revealed a significant correlation between FCHSD1 expression and tumor purity in 13 types of tumors, with a predominant negative correlation. The expression of FCHSD1 was positively correlated with the infiltration of six immune cells, including B cells, CD8T cells, CD4T cells, macrophages, neutrophils and dendritic cells. LGG, LIHC, and PRAD are the cancer phenotypes most associated with FCHSD1 expression of all tumors (**Figures 5A–U**). These results suggest that FCHSD1 may play a similar role in immune invasion in different cancer types.

**Figure 6:**
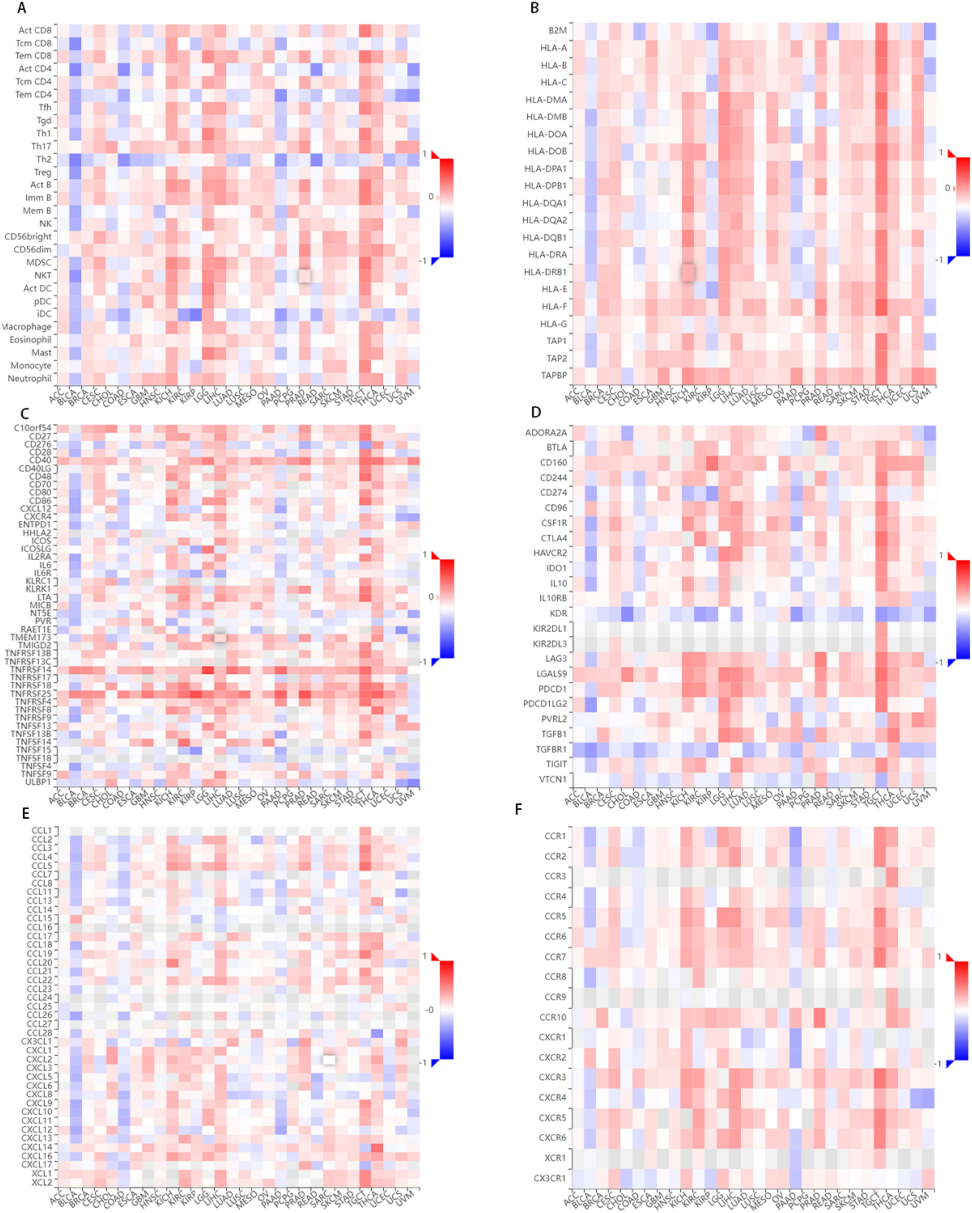
FCHSD1 expression is linked to immune infiltration as analyzed using the TISIDB database. FCHSD1 expression affected lymphocyte infiltration **(A)**, MHC expression **(B)**, immunostimulator expression **(C)**, immune inhibitors expression **(D)**, chemokine expression **(E)** and chemokine receptors expression **(F)** in human cancers.

The relationship between FCHSD1 and immune infiltration was further confirmed using the TISIDB database, which explored 28 infiltrating lymphocytes (**Figure 6A**). The expression of FCHSD1 in BRCA, KIRC, LGG, LIHC, LUAD and PRAD was positively correlated with the infiltration of activated CD8T cells and effector memory CD8T, while the infiltration of effector memory CD4T and effector memory CD4T was negatively correlated with the expression of FCHSD1. In these cancers, FCHSD1 expression was positively correlated with Th1 and Th17 infiltration in CD4T cell subsets, while FCHSD1 expression was negatively correlated with Th2 infiltration. Finally, infiltration of innate immune cells, including eosinophils and natural killer (NK) cells, was also positively correlated with FCHSD1 expression in LGG, LIHC, and PRAD. IDC is negatively correlated with FCHSD1 expression in LGG, LIHC, and PRAD. These results reveal the contrasting effects of FCHSD1 on adaptive and innate immune cell infiltration in different cancers.

We also investigated the correlation between FCHSD1 and the major histocompatibility complexes, immunosuppressants, immunostimulants, chemokines, and receptors expressed in the tumor microenvironment (TME) of these cancers (**Figures 6B–F**). The results showed that FCHSD1 had a positive effect on the expression of MHC class II molecules (HLA-DP, HLA-DM, HLA-DO, HLA-DQ, HLA-DR) in LGG, LIHC and PRAD. In LGG, LIHC, and PRAD, high expression of FCHSD1 positively affects the expression of LGALS9, TNFRSF14, TNFRSF25, but negatively affects the expression of KDR. These results show that the expression of FCHSD1 not only affects immune cell infiltration, but also affects the expression of major histocompatibility complexes (MHC) and immune cell activity by regulating the expression of immunosuppressants, chemokines/cytokines and their receptors.

### Analysis of the link between FCHSD1 expression level and prognosis

**Figure 7:**
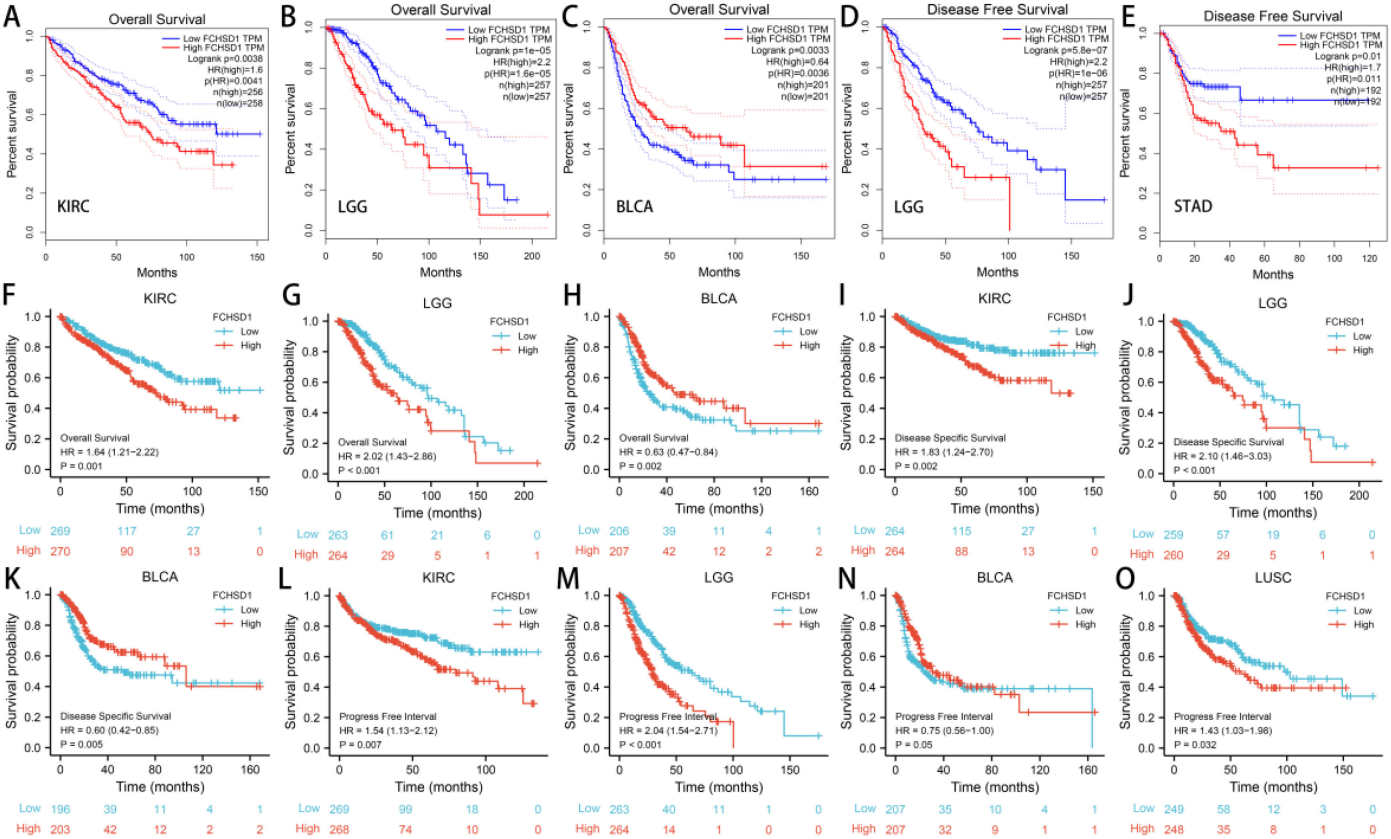
The GEPIA database and R package suggests that higher FCHSD1 expression correlates with worse prognosis in most cancers. In the GEPIA database, FCHSD1 expression was significantly correlated with overall survival of KIRC **(A)**, LGG **(B)**, BLCA **(C)**, and significantly correlated with disease-free survival of LGG **(D)**, STAD **(E)**. In the R package, FCHSD1 expression was significantly correlated with overall survival of KIRC **(F)**, LGG **(G)**, BLCA **(H)**, and significantly correlated with disease specific survival of KIRC **(I)**, LGG **(J)**, BLCA **(K)**. FCHSD1 expression was significantly correlated with progress free interval of KIRC **(L)**, LGG **(M)**, BLCA **(N)**, and LUSC **(O)**.

In order to investigate the relationship between FCHSD1 expression levels and the prognosis of cancer patients, we conducted survival correlation analysis on pan-cancer using several databases. Firstly, we utilized GEPIA to evaluate the impact of FCHSD1 expression levels on the prognosis of pan-cancer patients. The results indicated that higher FCHSD1 expression levels were associated with poorer overall survival (OS) in patients with KIRC (*p*=0.0038) and LGG (*p*=1e-05) (**Figures 7A, B**). Notably, FCHSD1 was identified as a protective factor for OS in BLCA patients (*p*=0.0033) (**Figure 7C**). Next, we explored the relationship between FCHSD1 expression levels and recurrence-free survival (RFS) (**Figures 7D, E**). High FCHSD1 expression levels were found to be associated with shorter RFS in cancers such as LGG (*p*=5.8e-07) and STAD (*p*=0.01).

Subsequently, we utilized R packages to establish the correlation between FCHSD1 expression and the prognosis of cancer patients. The data used in the R packages were derived from GEO, EGA, and TCGA databases. The results showed that high FCHSD1 expression was associated with shorter overall survival (OS) in cancer patients such as KIRC (*p*=0.001) and LGG (*p*<0.001), while increased FCHSD1 expression in BLCA (*p*=0.002) patients indicated prolonged OS (**Figures 7F–H**). Similarly, higher FCHSD1 expression was correlated with shorter disease-specific survival (DSS) in KIRC (*p*=0.002), and LGG (*p*<0.001) patients, whereas FCHSD1 acted as a protective factor for DSS in BLCA (*p*=0.005) patients (**Figures 7I–K**). For progression-free interval (PFI), the protective effect of FCHSD1 on BLCA (*p*=0.05) patients gradually turned into a detrimental factor over time (**Figure 7N**). High FCHSD1 expression was associated with shorter PFI in KIRC (*p*=0.007), LGG (*p*<0.001), LUSC (*p*=0.032) (**Figures 7 L, M, O**).

The results from both GEPIA and R packages indicate that the expression of FCHSD1 is significantly correlated with OS, DSS, and RFS in BLCA, KIRC, and LGG patients. In order to further elucidate the prognostic role of FCHSD1 in these cancers, a Cox proportional hazards regression model was established to evaluate the prognostic factors. These cancer patients were divided into high and low FCHSD1 expression groups based on the median expression of FCHSD1. Both univariate and multivariate Cox analyses revealed that high FCHSD1 expression is associated with poor prognosis in KIRC and LGG patients. Conversely, lower FCHSD1 expression is correlated with adverse prognosis in these patients.

**Figure 8:**
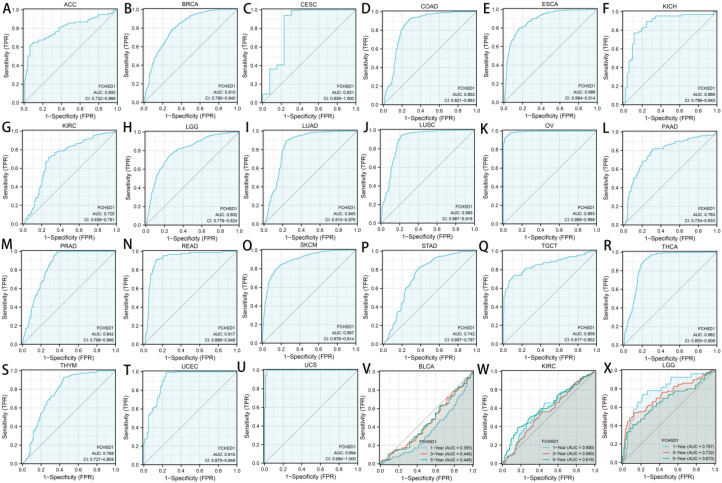
The diagnostic value of FCHSD1 for different cancers. **(A-U)** The receiver operating characteristic (ROC) and area under the curve (AUC) of FCHSD1 for pan-cancer. The time-dependent receiver operating characteristic (ROC) and area under the curve (AUC) of FCHSD1 for BLCA **(V)**, KIRC **(W)**, and LGG **(X)**.

We then used the receiver operating characteristic curve (ROC) to verify the diagnostic value of FCHSD1 for different cancers. FCHSD1 has high diagnostic accuracy (AUC greater than 0.7) for most cancers (**Figures 8A–U**). Furthermore, we utilized time-dependent ROC analysis to evaluate the diagnostic value of FCHSD1 in BLCA, KIRC, and LGG.

We further use time-dependent ROC analysis to demonstrate the diagnostic value of FCHSD1 for BLCA, KIRC, and LGG (**Figures 8V–X**). For the 1, 3, and 5-year OS of BLCA, the area under the curve (AUC) values are 0.71, 0.661, and 0.699, respectively. For the 1-year, 3-year, and 5-year OS of KIRC, the AUC values are 0.590, 0.560, and 0.616, respectively. For LGG’s 1-year, 3-year, and 5-year OS, the AUC values are 0.787, 0.732, and 0.670, respectively. The results showed that the expression of FCHSD1 had a strong predictive effect on the prognosis of BLCA, KIRC and LGG.

In conclusion, the results of survival curve and ROC curve analysis showed that FCHSD1 is a valuable diagnostic biomarker for many types of cancer, mainly including LGG, KIRC, BLCA.

### Analysis of FCHSD1 expression and cancer drug sensitivity in pan-cancer

**Figure 9:**
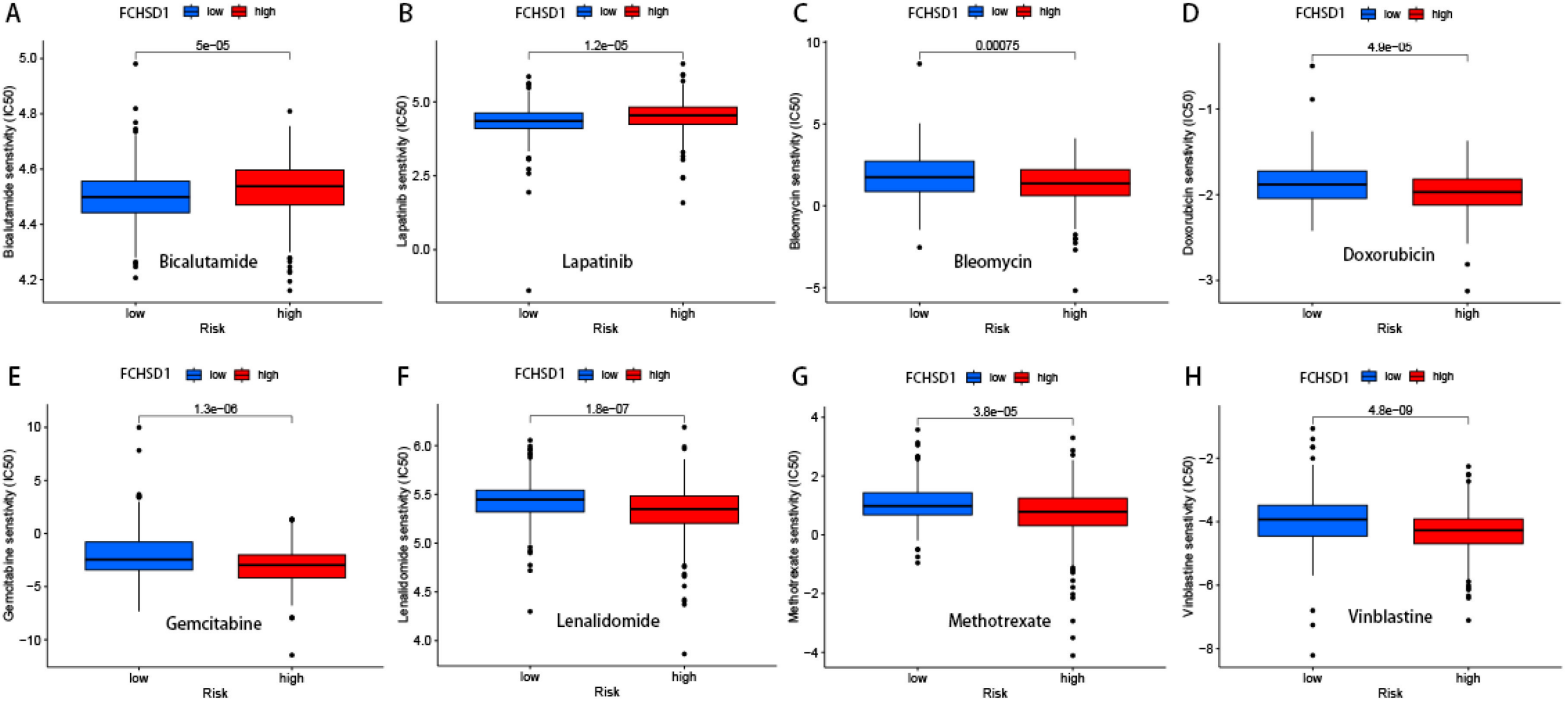
Drug sensitivity analysis of FCHSD1 using R package. The expression of FCHSD1 was associated with the sensitivity of Bicalutamide **(A)**, Lapatinib **(B)**, Bleomycin **(C)**, Doxorubicin **(D)**, Gemcitabine **(E)**, Lenalidomide **(F)**, Methotrexate **(G)**, Vinblastine **(H)**.

We delved deeper into the potential correlation between FCHSD1 expression and clinical drug sensitivity using R packages. Our findings revealed a positive correlation between FCHSD1 expression and the sensitivity of Bicalutamide and Lapatinib (**Figures 9A, B**). On the other hand, FCHSD1 expression showed a negative correlation with the sensitivity of drugs such as Bleomycin, Doxorubicin, Gemcitabine, Lenalidomide, Methotrexate, Nilotinib, and Vinblastine (**Figures 9C–H**). These data suggest that FCHSD1 may be associated with the chemoresistance of certain commonly used chemotherapy drugs in clinical practice, such as Gemcitabine and Vinblastine.

### Functional enrichment analysis reveals the role of FCHSD1

**Figure 10:**
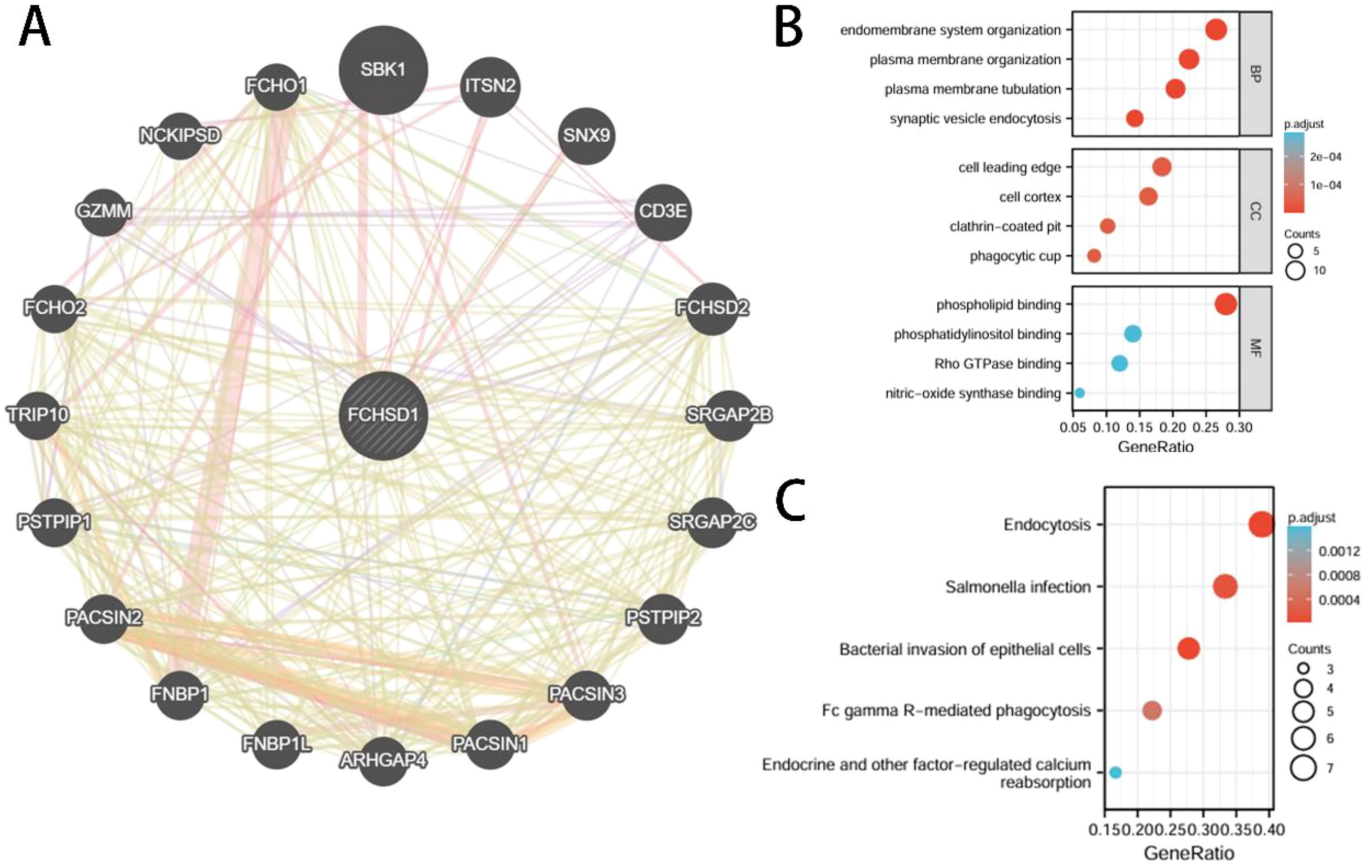
Interactive genes of FCHSD1 and gene ontology (GO) analysis. **(A)** FCHSD1 interacted with SBK1 and other genes in GeneMANIA database. **(B, C)** Top 50 interactive genes of FCHSD1 were obtained from STRING database and processed for GO enrichment analyses including biological process, cellular component, and molecular function and Kyoto Encyclopedia of Genes and Genomes (KEGG) analysis.

In order to delve deeper into the molecular mechanisms of FCHSD1 in tumor development, we screened for FCHSD1 binding proteins to conduct protein-protein interaction (PPI) network analysis. Using the GeneMANIA platform, we constructed a PPI network for FCHSD1, which revealed a strong interaction between FCHSD1 and the SH3 domain-binding kinase 1 (SBK1) (**Figure 10A**). Subsequently, we utilized both the STRING and GEPIA tools to identify 50 and 100 FCHSD1-related binding proteins, respectively. By performing a cross-analysis of these two sets, we identified two FCHSD1-related binding proteins, namely Intersectin 2 (ITSN2) and Formin-binding protein 4 (FNBP4). Both ITSN2 and FNBP4 exhibited a strong positive correlation with FCHSD1 expression. To further elucidate these findings, we conducted GO and KEGG enrichment analyses, which are presented below (**Figures 10B, C**). BP includes endomembrane system organization, plasma membrane organization, plasma membrane tubulation, synaptic vesicle endocytosis. CC includes cell leading edge, cell cortex, clathrin-coated pit, phagocytic cup. MF includes phospholipid binding, phosphatidylinositol binding, Rho GTPase binding, nitric-oxide synthase binding. KEGG includes endocytosis, Salmonella infection, bacterial invasion of epithelial cells, Fc gamma R-mediated phagocytosis, endocrine and other factor-regulated calcium reabsorption.

### *In vitro* experimental validation of FCHSD1 expression levels in cells and tissues

**Figure 11:**
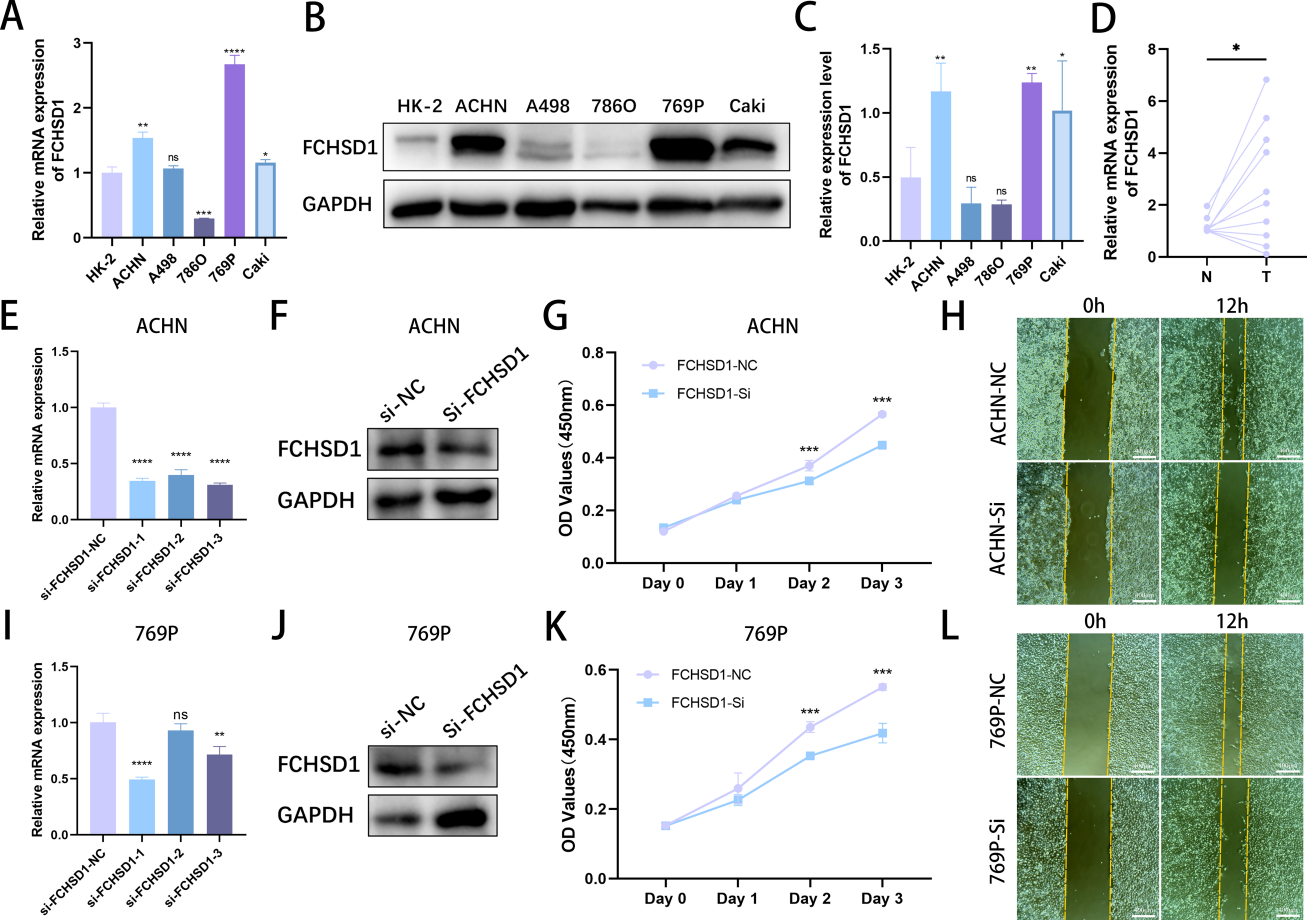
*In vitro* experimental investigation of FCHSD1 expression levels and their impact on the malignant progression of renal cancer. **(A)** qRT-PCR analysis of FCHSD1 mRNA expression levels in normal renal tubular cells and five renal cancer cell lines. **(B)** Western blot analysis of FCHSD1 protein expression levels in normal renal tubular cells and five renal cancer cell lines. **(C)** Quantitative analysis of western blot bands, n=3. **(D)** qRT-PCR analysis of FCHSD1 mRNA expression levels in adjacent non-cancerous tissues and renal cancer tissues. **(E)** qRT-PCR assessment of the silence efficiency of three siRNAs targeting FCHSD1 in ACHN cells. **(F)** Western blot verification of the silence efficiency of siRNA-1 targeting FCHSD1 in ACHN cells. **(G)** CCK8 assay to evaluate the effect of FCHSD1 knockdown on the proliferation of ACHN cells. **(H)** Wound healing assay to assess the impact of FCHSD1 knockdown on the migration of ACHN cells. **(I)** qRT-PCR assessment of the silence efficiency of three siRNAs targeting FCHSD1 in 769P cells. **(J)** Western blot verification of the silence efficiency of siRNA-1 targeting FCHSD1 in 769P cells. **(K)** CCK8 assay to evaluate the effect of FCHSD1 knockdown on the proliferation of 769P cells. **(L)** Wound healing assay to assess the impact of FCHSD1 knockdown on the migration of 769P cells.

To validate the results of the aforementioned bioinformatics analysis, we conducted qRT-qPCR using normal renal tubular cells (HK-2) and five renal cancer cell lines. The results indicated that, compared with HK-2 cells, the mRNA levels of FCHSD1 were significantly increased in ACHN, 769P, and Caki cells, whereas they were markedly decreased in 786O cells (**Figure 11A**). No significant difference in FCHSD1 mRNA expression was observed between HK-2 and A498 cells. Subsequently, we performed WB experiments on these cell lines, and the results were consistent with the qRT-PCR findings, showing that FCHSD1 protein levels were significantly elevated in ACHN, 769P, and Caki cells compared with HK-2 cells (**Figures 11B, C**). Furthermore, to further verify the expression of FCHSD1 in renal cancer patients, we conducted qRT-PCR on renal cancer tissues and adjacent non-cancerous tissues from multiple patients. The results revealed that FCHSD1 was highly expressed in renal cancer tissues compared with adjacent tissues (**Figure 11D**).

### Investigating the impact of FCHSD1 expression levels on the malignant progression of renal cancer

To investigate whether FCHSD1 promotes the malignant progression of renal cancer, three FCHSD1 siRNAs were transfected into ACHN and 769P cells. qRT-PCR was used to assess the silence efficiency of the three siRNAs on FCHSD1 in ACHN and 769P cells (**Figures 11E, I**). The results showed that si-FCHSD1–1 exhibited the highest efficiency in both cell lines. The aforementioned results were verified by western blot (**Figures 11F, J**). Therefore, si-FCHSD1–1 was used for subsequent experiments to silence FCHSD1. CCK-8 assays demonstrated that silence of FCHSD1 expression significantly reduced the proliferation rate of ACHN cells on the second and third days compared with cells transfected with si -NC (**Figure 11G**). Similarly, on the second and third days, silence of FCHSD1 expression markedly slowed the proliferation of 769P cells compared with si-NC-transfected cells (**Figure 11K**). Wound healing assays indicated that FCHSD1 silence significantly inhibited the wound healing ability of ACHN and 769P cells compared with si-NC-transfected cells (**Figures 11H, L**). Overall, downregulation of FCHSD1 expression can inhibit the malignant progression of renal cancer.

## Discussion

FCHSD1 is a member of the F-BAR family containing one amino terminal F-BAR domain and two SH3 domains (3, 4). Numerous studies have indicated that certain members of the F-BAR protein family are associated with the development of neurological disorders, chronic obstructive pulmonary disease (COPD), and tumors (9–12). However, there is no systematic analysis of FCHSD1 in pan-carcinoma. This study aims to provide a thorough analysis of FCHSD1 in pan-cancer, in order to reveal its potential role and underlying mechanisms in the development and clinical outcomes of various cancers.

Identifying aberrant gene expression in tumors is critical for discovering tumor-specific therapeutic targets (26). Previous studies have employed cancer xenograft models to further investigate this mechanisms (27). RNA sequencing has become an indispensable tool in contemporary cancer research. As one of the earliest research teams involved in TCGA biomarker studies, the Liu laboratory has substantially advanced the field through developing different strategies, notably: single gene for single cancer, gene set for single cancer, single gene pan-cancer, and gene set pan-cancer (28–44). The TIMER database contains high-throughput RNA sequencing data from 33 types of cancer from TCGA (17).We conducted mining analyses on cancer data from TCGA and GTEx databases using TIMER and R packages, respectively. TIMER results showed that FCHSD1 expression increased in 13 types of cancer. Further comparison using the TCGA + GTEx database with R packages revealed that FCHSD1 increased in 5 types of cancer. The reason for this difference may be due to the fact that the GTEx database contains more normal sample data. In summary, our study indicates that FCHSD1 mRNA expression is significantly increased in various cancer tissues compared with normal tissues. Additionally, CPTAC data from UALCAN can analyze protein expression in six types of cancer (45). FCHSD1 protein expression decreased in UCEC, OC, LIHC, and HNSC, but increased in KIRC, GBM, PAAD, and OV. The difference between FCHSD1 mRNA and protein expression may be attributed to the small sample size in the CPTAC dataset. We also evaluated the correlation between FCHSD1 expression and different cancer stage classifications. FCHSD1 plays different roles in the occurrence and development of different cancers, such as COAD, KIRC, BLCA, and LIHC. Therefore, FCHSD1 may be a potential therapeutic target or biomarker for multiple types of cancer.

We utilized the cBioPortal website to investigate the genetic alterations of FCHSD1 in human cancers and found that the major genetic alterations of FCHSD1 in cancers are mutations, structural variations, amplifications, and deep deletions. Specific gene mutations can predict the prognosis and treatment response of patients (46, 47). The highest frequency of change in FCHSD1 (> 6%) occurred in patients with KIRC, with “amplification” and “mutation” as the main types. Gene amplification is considered to be the main cause and typical manifestation of tumorigenesis (48). The main type of FCHSD1 gene alteration is missense mutation, and the important altered site is G559R. This is likely to be an important risk factor for tumorigenesis and a potential target for future cancer treatment. However, further research is needed.

TMB is a promising pan-cancer predictive biomarker that can guide precision immunotherapy (49, 50). In addition, TMB can also predict prognosis after immunotherapy in pan-cancer patients (51). TME features look for markers of tumor cell response to immunotherapy and influence clinical outcomes after treatment (52). Tumor infiltration of immune cells has an important impact on tumor initiation and progression, which can inhibit or promote tumor progression (53). Thorsson et al. integrated immunogenomics methods to use TCGA database data to establish six immune subtypes to partition human cancers, which are used to predict disease outcomes across multiple cancers (54). These six immune subtypes comprise: wound healing (C1), IFN-g dominant (C2), inflammatory (C3), lymphocyte depleted (C4), immunologically quiet (C5), and TGF-b dominant (C6). We explored the role of FCHSD1 expression in human cancer immunity and molecular subtypes through the TISIDB website. We note that FCHSD1 differs in immunoisoforms and molecular subtypes of different cancers and is the lowest expressed in human cancers with C4 and C5 immune subtypes. We further confirmed the relationship between FCHSD1 and immune infiltration using the TISIDB database, which explored the correlation between 28 infiltrating lymphocytes, major histocompatibility complexes, immunosuppressants, immunostimulants, chemokines, and receptors expressed in the tumor microenvironment (TME). For immune infiltrating cells, we found that immune cells such as Th2, macrophages and iDC were low infiltrate across most cancer types, while cells such as Th17 and MDSC showed high correlation. These results reveal the contrasting effects of FCHSD1 on adaptive and innate immune cell infiltration in different cancers. MDSCs have potent immunosuppressive activity and can promote tumor progression by promoting tumor cell survival, angiogenesis, invasion, and metastasis (55). MDSC infiltration levels are strongly correlated with clinical outcomes and therapeutic efficacy (56). Th17 cells have very high plasticity. Th17 cells acquire pathogenic activity by becoming Th2 cells during asthma or Th1 cells during infections, cancer, and autoimmune diseases (57). Studies have reported that Th17 cells are commonly associated with a variety of cancers such as lung, breast, prostate, colon and melanoma (58). This may indicate the immune mechanism of FCHSD1 on tumorigenesis and development. Studying the condition of tumor-infiltrating immune cells can lead to the development of strategies to intervene in the anti-tumor immune response. Using the TIMER database, we found that FCHSD1 expression was positively correlated with the infiltration of 6 immune cells. These results suggest that FCHSD1 may play a similar role in immune infiltration in different cancer types, leading to similar immune response protocols.

Through analysis with the GEPIA platform and R package, we discovered that enhanced expression of FCHSD1 is associated with poor prognosis in several cancers. It is noteworthy that FCHSD1 is a protective factor in BLCA. Cox proportional hazards regression confirmed the prognostic significance of FCHSD1 across multiple cancers. At the same time, we found through the ROC curve of cancer that the expression of FCHSD1 also has a strong predictive value for the prognosis of these cancers. These results support that the level of FCHSD1 expression affects the disease prognosis of patients. It is urgently needed to further elucidate the mechanism by which FCSHD1 affects the survival of different cancer patients.

To further investigate the molecular mechanism of FCHSD1 in tumorigenesis, we used the GeneMANIA platform to map a PPI network for FCHSD1 and found SBK1 with a strong interaction with FCHSD1. In addition, we intersected FCHSD1-related genes obtained from STRING and GEPIA2 and found two common genes, (ITSN2) and (FNBP4). SBK1 is a novel serine/threonine kinase named after its protein structure (59). Now it has been confirmed by a number of studies that it is related to fat metabolism and tumor occurrence and development, and can effectively predict cancer and treatment effects. ITSN-2L is a relatively conserved family of proteins. It acts as a guanine nucleotide exchanger for Cdc42 and regulates different steps of endocytosis in EC by controlling the Cdc42-WASp-Arp2/3 actin polymerization pathway (60). Formalin promotes the initiation and elongation of actin filaments and regulates cytoskeletal remodeling to participate in a variety of biological processes, such as cell division and endocytosis (61, 62). Studies have shown that its high expression promotes the occurrence and development of liver cancer (63). FCHSD1 may functionally interact with SBK1, ITSN2, and FNBP4 to collectively regulate malignant tumor progression. Nowadays, with the development of computational biology and bioinformatics, multiple computational methods, especially KEGG pathway and GO terminology, have been widely used to describe specific pathways and biological processes in different cancers (64, 65). In this study, 50 interacting genes and 100 related genes were obtained using STRING and GEPIA. The KEGG pathway and GO term methods were then used to perform functional enrichment analysis on 150 genes. Studies have shown that these genes may play an important role in “endocytosis”, “endometrial system organization”, “cell lead”, “phospholipid binding”, and other pathways. Endocytosis is the process by which cells actively internalize molecular and surface proteins through the inner membrane system and its membrane proteins and phospholipids (66). Cells use endocytosis to regulate signaling and acquire extracellular environmental changes to respond appropriately (67). Many studies have demonstrated that endocytosis may alter cancer cell proliferation, invasion, or metastasis by regulating receptor internalization, recycling, and degradation, as well as affecting cytoskeletal dynamics (67–70). In summary, targeting endocytosis mechanisms may be a viable and promising therapeutic strategy for cancer and metastasis, and FCHSD1 and related protein networks may be a viable entry point.

Furthermore, we validated the expression levels of FCHSD1 in normal renal tubular epithelial cells and five renal cancer cell lines, as well as in adjacent non-cancerous tissues and renal cancer tissues from patients with renal cancer through *in vitro* experiments. Both qRT-PCR and Western blot (WB) confirmed the upregulation of FCHSD1 expression in kidney renal clear cell carcinoma (KIRC) tissues and cells. To further explore the role of FCHSD1 in the malignant progression of renal cancer, we silenced its expression in cell lines. Cellular functional experiments were conducted on the silenced ACHN and 769P cell lines, revealing that FCHSD1 silencing inhibited the proliferation and migration of ACHN and 769P cells. These results corroborate the accuracy and reliability of the aforementioned bioinformatics analysis, and we plan to conduct similar and more in-depth molecular biology validations in additional cancers in the future.

We acknowledge that our study has several limitations. Firstly, the number of validations for FCHSD1 expression levels in tissues is relatively small, and we only validated FCHSD1 expression in renal cancer cells and tissues. We need to use different methods, such as immunohistochemistry, to verify FCHSD1 expression in various cancer tissues. Secondly, although FCHSD1 expression is correlated with immune responses and clinical survival in human malignancies, we are uncertain about how FCHSD1 affects the clinical survival of cancer patients through immune pathways. FCHSD1 may functionally cooperate with interacting genes (e.g., SBK1, ITSN2, and FNBP4) to collectively drive tumor malignancy, thus warranting further mechanistic investigations to elucidate these molecular interactions. Lastly, while comprehensive cancer genomics databases have emerged as invaluable research resources, significant analytical challenges remain due to inherent technical biases and biological variability (71, 72).

Our study systematically analyzed FCHSD1 in a pan-carcinogenic manner, evaluated the potential association of FCHSD1 expression with pathological stage, immunophenotype, immune cell infiltration, genetics, prognosis, drug sensitivity, etc. in various cancer types. In summary, upregulation of FCHSD1 expression predicts poor prognosis in multiple cancers. At the same time, FCHSD1 affects immune cell infiltration in some cancers. Therefore, FCHSD1 may serve as a valuable biomarker for cancer diagnosis, prognosis prediction, and immune infiltration assessment in human malignancies. Future research should prioritize the development of FCHSD1-targeted molecular therapies to suppress tumor progression, invasion, and metastasis across multiple cancer types.

## Conclusion

FCHSD1 has the potential to serve as a prognostic and immunological marker for pan-cancer, and may also be a crucial target for future immunotherapy.

## Data Availability

The original contributions presented in the study are included in the article/supplementary material. Further inquiries can be directed to the corresponding authors.

## References

[1] Siegel RL Giaquinto AN Jemal A. Cancer statistics, 2024. CA Cancer J Clin. (2024) 74:12–49. doi: 10.3322/caac.21820 38230766

[2] Sonkin D Thomas A Teicher BA. Cancer treatments: Past, present, and future. Cancer Genet. (2024) 286-287:18–24. doi: 10.1016/j.cancergen.2024.06.002 38909530 PMC11338712

[3] Katoh M Katoh M. Identification and characterization of human FCHSD1 and FCHSD2 genes in silico. Int J Mol Med. (2004) 13:749–54. doi: 10.3892/ijmm.13.5.749 15067381

[4] Greer P. Closing in on the biological functions of Fps/Fes and Fer. Nat Rev Mol Cell Biol. (2002) 3:278–89. doi: 10.1038/nrm783 11994747

[5] Itoh T Erdmann KS Roux A Habermann B Werner H De Camilli P. Dynamin and the actin cytoskeleton cooperatively regulate plasma membrane invagination by BAR and F-BAR proteins. Dev Cell. (2005) 9:791–804. doi: 10.1016/j.devcel.2005.11.005 16326391

[6] Takano K Toyooka K Suetsugu S. EFC/F-BAR proteins and the N-WASP-WIP complex induce membrane curvature-dependent actin polymerization. EMBO J. (2008) 27:2817–28. doi: 10.1038/emboj.2008.216 PMC2580791 18923421

[7] Kogata N Masuda M Kamioka Y Yamagishi A Endo A Okada M. Identification of Fer tyrosine kinase localized on microtubules as a platelet endothelial cell adhesion molecule-1 phosphorylating kinase in vascular endothelial cells. Mol Biol Cell. (2003) 14:3553–64. doi: 10.1091/mbc.e03-02-0080 PMC196549 12972546

[8] Chitu V Stanley ER. Pombe Cdc15 homology (PCH) proteins: coordinators of membrane-cytoskeletal interactions. Trends Cell Biol. (2007) 17:145–56. doi: 10.1016/j.tcb.2007.01.003 17296299

[9] Coyle IP Koh YH Lee WC Slind J Fergestad T Littleton JT. Nervous wreck, an SH3 adaptor protein that interacts with Wsp, regulates synaptic growth in Drosophila. Neuron. (2004) 41:521–34. doi: 10.1016/S0896-6273(04)00016-9 14980202

[10] Soderling SH Binns KL Wayman GA Davee SM Ong SH Pawson T. The WRP component of the WAVE-1 complex attenuates Rac-mediated signalling. Nat Cell Biol. (2002) 4:970–5. doi: 10.1038/ncb886 12447388

[11] Heath RJ Insall RH. Dictyostelium MEGAPs: F-BAR domain proteins that regulate motility and membrane tubulation in contractile vacuoles. J Cell Sci. (2008) 121:1054–64. doi: 10.1242/jcs.021113 18334553

[12] Han Y Cui J Lu Y Sue S Arpaia E Mak TW. FCHSD2 predicts response to chemotherapy in acute myeloid leukemia patients. Leuk Res. (2012) 36:1339–46. doi: 10.1016/j.leukres.2012.06.011 22902056

[13] Kim SJ Khadka D Seo JH. Interplay between solid tumors and tumor microenvironment. Front Immunol. (2022) 13:882718. doi: 10.3389/fimmu.2022.882718 35707536 PMC9189309

[14] Chen SMY Krinsky AL Woolaver RA Wang X Chen Z Wang JH. Tumor immune microenvironment in head and neck cancers. Mol Carcinog. (2020) 59:766–74. doi: 10.1002/mc.23162 PMC7282929 32017286

[15] Lv B Wang Y Ma D Cheng W Liu J Yong T. Immunotherapy: reshape the tumor immune microenvironment. Front Immunol. (2022) 13:844142. doi: 10.3389/fimmu.2022.844142 35874717 PMC9299092

[16] Darvin P Toor SM Sasidharan Nair V Elkord E. Immune checkpoint inhibitors: recent progress and potential biomarkers. Exp Mol Med. (2018) 50:1–11. doi: 10.1038/s12276-018-0191-1 PMC6292890 30546008

[17] Li T Fan J Wang B Traugh N Chen Q Liu JS. TIMER: A web server for comprehensive analysis of tumor-infiltrating immune cells. Cancer Res. (2017) 77:e108–10. doi: 10.1158/0008-5472.CAN-17-0307 PMC6042652 29092952

[18] Chandrashekar DS Bashel B Balasubramanya SAH Creighton CJ Ponce-Rodriguez I Chakravarthi BVSK. UALCAN: A portal for facilitating tumor subgroup gene expression and survival analyses. Neoplasia. (2017) 19:649–58. doi: 10.1016/j.neo.2017.05.002 PMC5516091 28732212

[19] Tang Z Li C Kang B Gao G Li C Zhang Z. GEPIA: a web server for cancer and normal gene expression profiling and interactive analyses. Nucleic Acids Res. (2017) 45:W98–w102. doi: 10.1093/nar/gkx247 28407145 PMC5570223

[20] Cerami E Gao J Dogrusoz U Gross BE Sumer SO Aksoy BA. The cBio cancer genomics portal: an open platform for exploring multidimensional cancer genomics data. Cancer Discovery. (2012) 2:401–4. doi: 10.1158/2159-8290.CD-12-0095 PMC3956037 22588877

[21] Gao J Aksoy BA Dogrusoz U Dresdner G Gross B Sumer SO. Integrative analysis of complex cancer genomics and clinical profiles using the cBioPortal. Sci Signal. (2013) 6:pl1. doi: 10.1126/scisignal.2004088 23550210 PMC4160307

[22] Szklarczyk D Gable AL Nastou KC Lyon D Kirsch R Pyysalo S. The STRING database in 2021: customizable protein-protein networks, and functional characterization of user-uploaded gene/measurement sets. Nucleic Acids Res. (2021) 49:D605–d612. doi: 10.1093/nar/gkaa1074 33237311 PMC7779004

[23] von Mering C Huynen M Jaeggi D Schmidt S Bork P Snel B. STRING: a database of predicted functional associations between proteins. Nucleic Acids Res. (2003) 31:258–61. doi: 10.1093/nar/gkg034 PMC165481 12519996

[24] Warde-Farley D Donaldson SL Comes O Zuberi K Badrawi R Chao P. The GeneMANIA prediction server: biological network integration for gene prioritization and predicting gene function. Nucleic Acids Res. (2010) 38:W214–20. doi: 10.1093/nar/gkq537 PMC2896186 20576703

[25] Ru B Wong CN Tong Y Zhong JY Zhong SSW Wu WC. TISIDB: an integrated repository portal for tumor-immune system interactions. Bioinformatics. (2019) 35:4200–2. doi: 10.1093/bioinformatics/btz210 30903160

[26] Andre F Mardis E Salm M Soria JC Siu LL Swanton C. Prioritizing targets for precision cancer medicine. Ann Oncol. (2014) 25:2295–303. doi: 10.1093/annonc/mdu478 25344359

[27] Li R Huang Y Liu H Dilger JP Lin J. Comparing volatile and intravenous anesthetics in a mouse model of breast cancer metastasis. Cancer Res. (2018) 78. doi: 10.1158/1538-7445.AM2018-2162

[28] Liu H Weng J Huang CL Jackson AP. Is the voltage-gated sodium channel β3 subunit (SCN3B) a biomarker for glioma? Funct Integr Genomics. (2024) 24:162. doi: 10.1007/s10142-024-01443-7 39289188

[29] Li Y Liu H. Clinical powers of Aminoacyl tRNA Synthetase Complex Interacting Multifunctional Protein 1 (AIMP1) for head-neck squamous cell carcinoma. Cancer biomark. (2022) 34:359–74. doi: 10.3233/CBM-210340 PMC12364190 35068446

[30] Liu H Li Y. Potential roles of Cornichon Family AMPA Receptor Auxiliary Protein 4 (CNIH4) in head and neck squamous cell carcinoma. Cancer biomark. (2022) 35:439–50. doi: 10.3233/CBM-220143 PMC12364253 36404537

[31] Liu H Weng J. A comprehensive bioinformatic analysis of cyclin-dependent kinase 2 (CDK2) in glioma. Gene. (2022) 822:146325. doi: 10.1016/j.gene.2022.146325 35183683

[32] Chhatwal KS Liu H. RAD50 is a potential biomarker for breast cancer diagnosis and prognosis. (2024). doi: 10.1101/2024.09.07.611821

[33] Liu H. Expression and potential immune involvement of cuproptosis in kidney renal clear cell carcinoma. Cancer Genet. (2023) 274-275:21–5. doi: 10.1016/j.cancergen.2023.03.002 36963335

[34] Liu H Tang T. A bioinformatic study of IGFBPs in glioma regarding their diagnostic, prognostic, and therapeutic prediction value. Am J Transl Res. (2023) 15:2140–55. PMC10086936 37056850

[35] Liu H Dong A Rasteh AM Wang P Weng J. Identification of the novel exhausted T cell CD8 + markers in breast cancer. Sci Rep. (2024) 14:19142. doi: 10.1038/s41598-024-70184-1 39160211 PMC11333736

[36] Liu H Dilger JP Lin J. A pan-cancer-bioinformatic-based literature review of TRPM7 in cancers. Pharmacol Ther. (2022) 240:108302. doi: 10.1016/j.pharmthera.2022.108302 36332746

[37] Liu H Weng J. A pan-cancer bioinformatic analysis of RAD51 regarding the values for diagnosis, prognosis, and therapeutic prediction. Front Oncol. (2022) 12:858756. doi: 10.3389/fonc.2022.858756 35359409 PMC8960930

[38] Liu H Karsidag M Chhatwal K Wang P Tang T. Single-cell and bulk RNA sequencing analysis reveals CENPA as a potential biomarker and therapeutic target in cancers. PloS One. (2025) 20:e0314745. doi: 10.1371/journal.pone.0314745 39820192 PMC11737691

[39] Liu H Weng J Huang CL Jackson AP. Voltage-gated sodium channels in cancers. biomark Res. (2024) 12:70. doi: 10.1186/s40364-024-00620-x 39060933 PMC11282680

[40] Liu H. Pan-cancer profiles of the cuproptosis gene set. Am J Cancer Res. (2022) 12:4074–81. doi: 10.21203/rs.3.rs-1716214/v1 PMC9442004 36119826

[41] Liu H Tang T. Pan-cancer genetic analysis of cuproptosis and copper metabolism-related gene set. Front Oncol. (2022) 12:952290. doi: 10.3389/fonc.2022.952290 36276096 PMC9582932

[42] Liu H Tang T. Pan-cancer genetic analysis of disulfidptosis-related gene set. Cancer Genet. (2023) 278-279:91–103. doi: 10.1016/j.cancergen.2023.10.001 37879141

[43] Agarwal K Liu H. Potential cancer biomarkers: mitotic intra-S DNA damage checkpoint genes. bioRxiv (2024).

[44] Dong A Rasteh AM Liu H. Pan-cancer genetic analysis of mitochondrial DNA repair gene set. bioRxiv (2024).

[45] Chen F Chandrashekar DS Varambally S Creighton CJ. Pan-cancer molecular subtypes revealed by mass-spectrometry-based proteomic characterization of more than 500 human cancers. Nat Commun. (2019) 10:5679. doi: 10.1038/s41467-019-13528-0 31831737 PMC6908580

[46] Sanz-Garcia E Argiles G Elez E Tabernero J. BRAF mutant colorectal cancer: prognosis, treatment, and new perspectives. Ann Oncol. (2017) 28:2648–57. doi: 10.1093/annonc/mdx401 29045527

[47] Allegra CJ Rumble RB Hamilton SR Mangu PB Roach N Hantel A. Extended RAS gene mutation testing in metastatic colorectal carcinoma to predict response to anti-epidermal growth factor receptor monoclonal antibody therapy: American society of clinical oncology provisional clinical opinion update 2015. J Clin Oncol. (2016) 34:179–85. doi: 10.1200/JCO.2015.63.9674 26438111

[48] Matsui A Ihara T Suda H Mikami H Semba K. Gene amplification: mechanisms and involvement in cancer. Biomol Concepts. (2013) 4:567–82. doi: 10.1515/bmc-2013-0026 25436757

[49] Fumet JD Truntzer C Yarchoan M Ghiringhelli F. Tumour mutational burden as a biomarker for immunotherapy: Current data and emerging concepts. Eur J Cancer. (2020) 131:40–50. doi: 10.1016/j.ejca.2020.02.038 32278982 PMC9473693

[50] Steuer CE Ramalingam SS. Tumor mutation burden: leading immunotherapy to the era of precision medicine? J Clin Oncol. (2018) 36:631–2. doi: 10.1200/JCO.2017.76.8770 29337637

[51] Samstein RM Lee CH Shoushtari AN Hellmann MD Shen R Janjigian YY. Tumor mutational load predicts survival after immunotherapy across multiple cancer types. Nat Genet. (2019) 51:202–6. doi: 10.1038/s41588-018-0312-8 PMC6365097 30643254

[52] Wu T Dai Y. Tumor microenvironment and therapeutic response. Cancer Lett. (2017) 387:61–8. doi: 10.1016/j.canlet.2016.01.043 26845449

[53] Lei X Lei Y Li JK Du WX Li RG Yang J. Immune cells within the tumor microenvironment: Biological functions and roles in cancer immunotherapy. Cancer Lett. (2020) 470:126–33. doi: 10.1016/j.canlet.2019.11.009 31730903

[54] Thorsson V Gibbs DL Brown SD Wolf D Bortone DS Ou Yang TH. The immune landscape of cancer. Immunity. (2019) 51:411–2. doi: 10.1016/j.immuni.2019.08.004 31433971

[55] Condamine T Ramachandran I Youn JI Gabrilovich DI. Regulation of tumor metastasis by myeloid-derived suppressor cells. Annu Rev Med. (2015) 66:97–110. doi: 10.1146/annurev-med-051013-052304 25341012 PMC4324727

[56] Zhang S Ma X Zhu C Liu L Wang G Yuan X. The role of myeloid-derived suppressor cells in patients with solid tumors: A meta-analysis. PloS One. (2016) 11:e0164514. doi: 10.1371/journal.pone.0164514 27780254 PMC5079654

[57] Guéry L Hugues S. Th17 cell plasticity and functions in cancer immunity. BioMed Res Int. (2015) 2015:314620. doi: 10.1155/2015/314620 26583099 PMC4637016

[58] Shahid A Bharadwaj M. The connection between the Th17 cell related cytokines and cancer stem cells in cancer: Novel therapeutic targets. Immunol Lett. (2019) 213:9–20. doi: 10.1016/j.imlet.2019.07.001 31278971

[59] Nara K Akasako Y Matsuda Y Fukazawa Y Iwashita S Kataoka M. Cloning and characterization of a novel serine/threonine protein kinase gene expressed predominantly in developing brain. Eur J Biochem. (2001) 268:2642–51. doi: 10.1046/j.1432-1327.2001.02157.x 11322885

[60] Klein IK Predescu DN Sharma T Knezevic I Malik AB Predescu S. Intersectin-2L regulates caveola endocytosis secondary to Cdc42-mediated actin polymerization. J Biol Chem. (2009) 284:25953–61. doi: 10.1074/jbc.M109.035071 PMC2757996 19622753

[61] Faix J Grosse R. Staying in shape with formins. Dev Cell. (2006) 10:693–706. doi: 10.1016/j.devcel.2006.05.001 16740473

[62] Ahangar P Cowin AJ. Reforming the barrier: the role of formins in wound repair. Cells. (2022) 11:2779. doi: 10.3390/cells11182779 36139355 PMC9496773

[63] Zheng KW Zhang CH Wu W Zhu Z Gong JP Li CM. FNBP4 is a potential biomarker associated with cuproptosis and promotes tumor progression in hepatocellular carcinoma. Int J Gen Med. (2023) 16:467–80. doi: 10.2147/IJGM.S395881 PMC9907010 36760683

[64] Kanehisa M Sato Y Kawashima M Furumichi M Tanabe M. KEGG as a reference resource for gene and protein annotation. Nucleic Acids Res. (2016) 44:D457–62. doi: 10.1093/nar/gkv1070 PMC4702792 26476454

[65] Ashburner M Ball CA Blake JA Botstein D Butler H Cherry JM. Gene ontology: tool for the unification of biology. The Gene Ontology Consortium. Nat Genet. (2000) 25:25–9. doi: 10.1038/75556 PMC3037419 10802651

[66] Khan I Steeg PS. Endocytosis: a pivotal pathway for regulating metastasis. Br J Cancer. (2021) 124:66–75. doi: 10.1038/s41416-020-01179-8 33262521 PMC7782782

[67] Yang XZ Li XX Zhang YJ Rodriguez-Rodriguez L Xiang MQ Wang HY. Rab1 in cell signaling, cancer and other diseases. Oncogene. (2016) 35:5699–704. doi: 10.1038/onc.2016.81 PMC5396462 27041585

[68] Chen PH Bendris N Hsiao Y-J Chen H-Y Yu S-L Schmid SL. Crosstalk between CLCb/Dyn1-mediated adaptive clathrin-mediated endocytosis and epidermal growth factor receptor signaling increases metastasis. Dev Cell. (2017) 40:278–88.e5. doi: 10.1016/j.devcel.2017.01.007 28171750 PMC5740869

[69] Joshi B Strugnell SS Goetz JG Kojic LD Cox ME Griffith OL. Phosphorylated caveolin-1 regulates Rho/ROCK-dependent focal adhesion dynamics and tumor cell migration and invasion. Cancer Res. (2008) 68:8210–20. doi: 10.1158/0008-5472.CAN-08-0343 18922892

[70] Boulay PL Schlienger S Lewis-Saravalli S Vitale N Ferbeyre G Claing A. ARF1 controls proliferation of breast cancer cells by regulating the retinoblastoma protein. Oncogene. (2011) 30:3846–61. doi: 10.1038/onc.2011.100 21478909

[71] Liu H Guo Z Wang P. Genetic expression in cancer research: Challenges and complexity. Gene Rep. (2024) 37. doi: 10.1016/j.genrep.2024.102042

[72] Liu H Li Y Karsidag M Tu T Wang P. Technical and biological biases in bulk transcriptomic data mining for cancer research. J Cancer (2025) 16(1):34–43. 39744578 10.7150/jca.100922 PMC11660120

